# Protein pyruvylation: a potential metabolic checkpoint in tumor immunity?

**DOI:** 10.3389/fimmu.2026.1827786

**Published:** 2026-10-19

**Authors:** Ren Liu, Jiamin Yuan, Haigang Jin, Lida Ge, Chunxia Dai, Wei Wang, Pengping Li, Zhenyu Wang

**Affiliations:** 1 Zhejiang Chinese Medical University, Hangzhou, Zhejiang, China; 2 Affiliated Xiaoshan Hospital, Hangzhou Normal University, Hangzhou, Zhejiang, China; 3 The Xiaoshan Affiliated Hospital of Wenzhou Medical University, The First People’s Hospital of Xiaoshan District Hangzhou, Hangzhou, Zhejiang, China; 4 Department of General Surgery, Nanyang Street Community Health Service Center, Xiaoshan, Hangzhou, China; 5 Department of Tumor Surgery, The Second Affiliated Hospital of Bengbu Medical University, Bengbu, Anhui, China

**Keywords:** IFN-I pathway, immunotherapy resistance, metabolism, protein pyruvylation, tumor immunity

## Introduction

Tumor aerobic glycolysis leads to pyruvate buildup in the tumor microenvironment (TME). Recently, STAT1 pyruvylation induced by PKM2 (pyruvate kinase M2) and pyruvate was shown to suppress type I interferon (IFN-I) signaling. In cancer immunotherapy, IFN-I shows a clear acute-versus-chronic paradox. Short-term IFN-I signaling helps antigen presentation and CD8^+^ T-cell activation. However, long-term IFN-I exposure promotes CD8^+^ T-cell exhaustion and reduces the benefit of checkpoint blockade. By linking pyruvate to the IFN-I pathway, pyruvylation may help explain this poor clinical response. We hypothesize that lasting pyruvate exposure in the TME keeps STAT1 pyruvylation high in infiltrating immune cells. This may silence endogenous IFN-I signaling for a long time and support tumor immune escape.

## Novel post-translational modification

Zuo et al. recently reported a new post-translational modification (PTM). In this process, pyruvate directly modifies amino acid residues. They named it protein pyruvylation (1). They showed that high glucose promotes STAT1 pyruvylation at Lys201. This modification stops STAT1 from binding STAT2. As a result, IFN-I signaling is suppressed and antiviral responses are weakened.

## IFN-I plays a dual role in tumor immune regulation

IFN-I is widely studied, but it shows a basic clinical paradox in tumor immunotherapy. In preclinical studies, acute IFN-I signaling helps dendritic cells present tumor antigens and activate cytotoxic CD8^+^ T cells. However, systemic IFN-I treatment often gives low response rates. This opposite phenotype may be related to low-dose IFN-I signaling and microenvironment reprogramming in human solid cancers (2). In addition, chronic IFN-I exposure can promote immunosuppression. It drives lipid-peroxidation-related terminal exhaustion of CD8^+^ T cells. It also upregulates transcription factors that form strong inhibitory feedback loops (3, 4).

## Pyruvate drives T-cell dysfunction and immune escape

Aerobic glycolysis (Warburg effect) leads to high pyruvate and lactate in the TME. Lactate can regulate tumor immunity through lactylation (5). Pyruvate can also support tumor metabolism and immune escape. It has been reported that tumor cells can change pyruvate use and succinate signaling in tumor-infiltrating CD8^+^ T cells by remodeling the local metabolic network. This weakens antitumor cytotoxicity (6). Moreover, pyruvate flux inside T cells also shapes their function. Mitochondrial pyruvate carriers (MPCs) are key regulators of T-cell fate. Short-term MPC inhibition can favor memory T-cell formation through a metabolic–epigenetic axis. In the nutrient-poor TME, effector T cells need MPCs to convert lactate into pyruvate for oxidation to keep antitumor activity (7).

## Clinical evidence gap and indirect support

To date, no immunotherapy cohort has directly measured STAT1 pyruvylation, or global lysine pyruvylation, together with IFN-I treatment response or immune-checkpoint response. The key finding that STAT1-K201 pyruvylation suppresses IFN-I signaling came from antiviral and high-glucose studies, not from cancer trial samples (1). However, several lines of evidence make a clinical link biologically reasonable. First, chronic IFN-I signaling drives terminal CD8^+^ T-cell exhaustion linked to lipid peroxidation and reduces anti-PD-1 efficacy. This gives a practical definition of IFN-related immunotherapy resistance (2–4). Second, tumor-driven changes in pyruvate use weaken CD8^+^ T-cell killing, and mitochondrial pyruvate handling shapes antitumor T-cell fate (6, 7). Pyruvate can also regulate PD-L1 and suppress T-cell immunity (8). Third, another metabolite-driven STAT1 change, lactylation at K193, weakens IFN-gamma-related chemokine programs and helps immune escape. Blocking this change can improve checkpoint blockade in preclinical models (9). Fourth, STAT1 pathway proteins have already been measured in melanoma cohorts treated with PD-1 blockade. Fifth, pyruvylation regulators themselves have an effect on the prognosis of IBC-treated real-world cohort, in which high expression of PKM or HAT1 has worse overall survival in melanoma with anti-PD-1 therapy, respectively, while high expression of SIRT3 has a better overall survival in melanoma with anti-PD-L1 therapy (**Figure 1**). This shows that IFN/STAT1 pathway status can be tested in patients, even though pyruvylation itself has not been measured yet (10). Therefore, whether IFN-I-related immunotherapy resistance is caused by pyruvylation is still unproven. Current data support a testable working model, not a cohort-proven causal claim. Priority studies include (i) targeted mass spectrometry of STAT1-K201 pyruvylation in pre- and on-treatment ICB biopsies; (ii) linking glycolytic/pyruvate gene and metabolite scores with IFN-stimulated gene programs and clinical response; and (iii) comparing pyruvylation levels between responders and non-responders.

**Figure 1:**
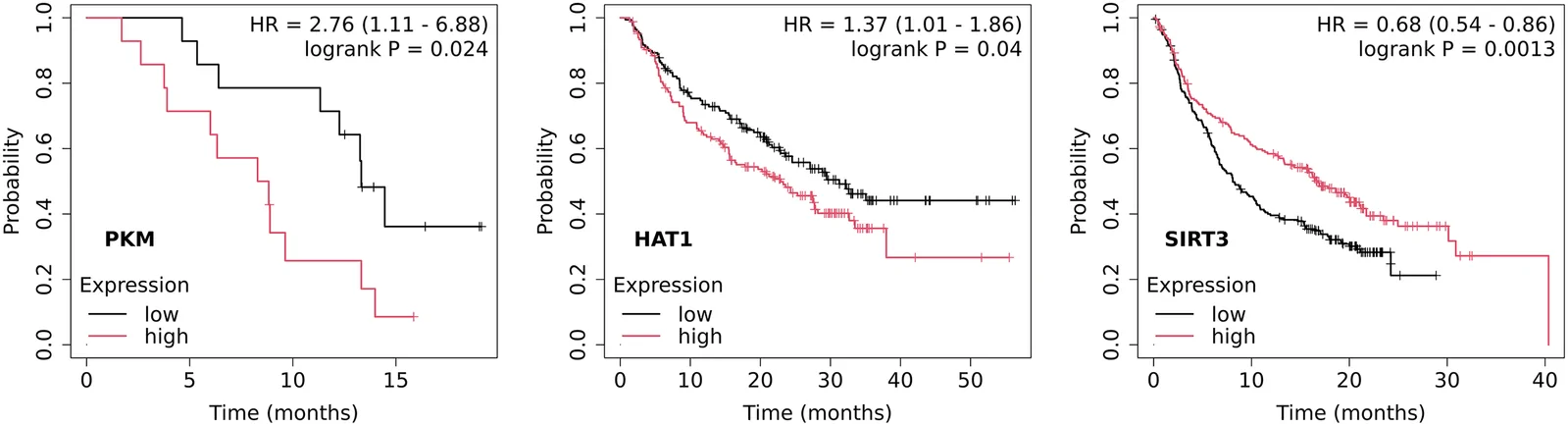
The prognostic effect of pyruvylation pivotal regulators in real-world cohorts. High expression of PKM or HAT1 has worse overall survival in melanoma with anti-PD-1 therapy, respectively, while high expression of SIRT3 has better overall survival in melanoma with anti-PD-L1 therapy. All data from the Kaplan–Meier Plotter: https://kmplot.com/analysis/index.php?p=home.

## Pyruvylation as a candidate mediator of IFN- and pyruvate-linked tumor immunity

Systemic IFN-I often shows limited antitumor effects, which differs from many preclinical findings. We propose that high IFN-I activity in a pyruvate-rich TME may trigger feedback inhibition of STAT1/STAT2 heterodimer formation through STAT1 pyruvylation. Here, we do not use the uncommon term “immunotherapy fatigue”. Instead, we mean early or lasting poor clinical response that reflects chronic IFN-I-driven CD8^+^ T-cell exhaustion and inhibitory feedback, not a separate formal diagnosis (2–4). At the whole-body level, pyruvate levels and STAT1 pyruvylation can change with glucose supply (1). However, in the highly glycolytic TME, pyruvate stays high. We hypothesize that this metabolic state keeps STAT1 pyruvylation high in tumor-infiltrating immune cells, weakens endogenous IFN-I signaling, and contributes to immunotherapy resistance.

## Future research suggestions

Future studies should measure protein pyruvylation in tumor-infiltrating lymphocytes and in paired ICB samples. In addition, the enzymes that control lysine pyruvylation still need testing in disease models. A recent systematic study of lysine pyruvylation identified HAT1 and p300 as candidate writers and SIRT3 as a depyruvylase (eraser). Kpy levels changed with glycolytic flux and pyruvate supply (11). These findings give a clear molecular starting point. However, it is still unknown whether SIRT3, or other erasers, remove STAT1-K201 pyruvylation in TIL subsets and restore IFN-I activity in tumors. Support for studying eraser enzymes also comes from lactylation research. After lactylation was reported, HDAC1–3 and some sirtuins were shown to act as delactylases. This shows that metabolite-driven lysine acylation is often reversible and enzyme-controlled (12, 13). For methods, we suggest (i) *in vitro* depyruvylation assays with pyruvylated STAT1 peptides or purified STAT1 plus recombinant SIRT3 and related deacylases; (ii) gain or loss of SIRT3, HAT1, and p300 in IFN-responsive immune and tumor cells, followed by STAT1–STAT2 binding, ISG induction, and IFN reporter assays; (iii) compartment-resolved proteomics to compare mitochondrial versus nuclear or cytosolic control of Kpy; and (iv) combined metabolic treatment and checkpoint blockade in models grouped by pyruvate or Kpy level. Collectively, clarifying writer and eraser specificity for STAT1 will help determine whether pyruvylation is a useful metabolic checkpoint in tumor immunity. Finally, we thank Zuo et al. for their important work, which opens a new direction in immunometabolism.
